# The Effect of Methylated Vitamin B Complex on Depressive and Anxiety Symptoms and Quality of Life in Adults with Depression

**DOI:** 10.1155/2013/621453

**Published:** 2013-01-21

**Authors:** John E. Lewis, Eduard Tiozzo, Angelica B. Melillo, Susanna Leonard, Lawrence Chen, Armando Mendez, Judi M. Woolger, Janet Konefal

**Affiliations:** ^1^Department of Psychiatry & Behavioral Sciences, Miller School of Medicine, University of Miami, Miami, FL 33136, USA; ^2^Department of Medicine, Miller School of Medicine, University of Miami, Miami, FL 33136, USA

## Abstract

Depression, the most common type of mental illness, is the second leading cause of disability and is increasing among Americans. The effect of improved nutrition, particularly with dietary supplements, on depression may provide an alternative to standard medical treatment. Some studies have shown that certain nutrients (e.g., inositol and S-adenosyl methionine) are effective at improving depressed mood, although the results are not unequivocal. The current study was a randomized, double-blind, placebo-controlled trial to evaluate the efficacy of a vitamin B complex nutritional supplement (Max Stress B) for improving depressive and anxiety symptoms according to the Beck Depression and Anxiety Inventories (BDI and BAI) in 60 adults diagnosed with major depression or other forms of depressive disorders. Secondary outcomes included quality of life according to the SF-36. Participants were assessed at baseline and 30- and 60-day followups. Max Stress B showed significant and more continuous improvements in depressive and anxiety symptoms, compared to placebo. Additionally, Max Stress B showed significant improvement on the mental health scale of the SF-36 compared to placebo. Thus, we showed modest utility of Max Stress B to improve mood symptoms and mental health quality of life in adults with depression.

## 1. Introduction

Approximately 26% of American adults are suffering from a diagnosable mental disease, and nearly half (45%) of them meet criteria for two or more disorders strongly related to comorbidity [1]. Major depressive disorder (MDD), one of the most common mental disorders, affects almost 15 million American adults (about 7% of the population) [1], and women report more depressive symptoms than men [2]. Furthermore, MDD is the leading cause of disability in the United States for persons between the ages of 15 and 44 [3], resulting in almost half of all lost productivity that translates into a cost burden of $44 billion per year [4]. In addition, the World Health Organization's Global Burden of Disease Study measured lost years of healthy life in the developed world, regardless of whether they were lost to premature death or disability for various diseases [5], concluding that disability burden caused by MDD ranks second only to cardiovascular disease.

While 35–45% of depressed patients receiving FDA-approved antidepressants experience complete relief from their symptoms, 55–65% have inadequate response and/or side effects, such as sexual dysfunction, insomnia, weight gain, restlessness, and memory lapses, among others [6]. Moreover, antidepressants have also been found to have serious side effects, such as suicide, violence, psychosis, and abnormal bleeding [7, 8]. Thus, many patients end up on revolving medication trials, switching repeatedly from one drug to another or combining drugs to maximize their effects. In addition, a literature review found that among patients with MDD placebo was as effective as antidepressants [9].

Interestingly, according to several studies, modifiable factors, such as optimizing nutritional status, may help to improve the symptoms of depression [10]. In one study, 12 g/day of inositol resulted in positive therapeutic improvements similar to common antidepressant drugs, but without untoward side effects [11]. Additional research has also confirmed the positive value of inositol for treating depressive symptoms [12]. Ernst found that Ginkgo biloba was effective at improving depressed mood, anxiety, memory, concentration, and fatigue [13]. S-Adenosyl Methionine (SAMe) was compared to oral doses of Imipramine in a double-blind study for 14 days. Significant improvements were observed with SAMe by the end of the first week, and at the end of the protocol 66% of the SAMe patients had a clinically significant improvement in depressive symptoms compared to 22% of the Imipramine patients [14]. Another study that included 23 elderly patients with type 2 diabetes and hypomagnesemia showed that magnesium chloride was as effective as 50 mg/day of Imipramine in improving depressive symptoms [15]. Given these positive findings, a low-cost, safe alternative to medication can be considered for persons suffering from MDD.

Thus, our study will extend the evaluative process of a vitamin B complex supplement's efficacy and safety in improving depression, anxiety, and quality of life in a sample of adults diagnosed with depression through a randomized, double-blind, placebo-controlled clinical trial. With the overall high prevalence of dietary supplement use, the multifaceted problems associated with depression, including the untoward effects of standard treatment, and the growing number of readily available alternative and complementary remedies in the United States, the efficacy and safety of these substances demand more randomized clinical studies. Therefore, only scientifically valid results from well-controlled trials can help to evaluate and support claims of effectiveness of all treatments, including natural products.

## 2. Methods

### 2.1. Study Participants

The study was conducted with the approval of the University of Miami Institutional Review Board for human subjects research, and each subject signed informed consent and HIPAA forms before enrolling in the study. Potential participants (*n* = 120) were identified through referrals from clinical offices and centers at the University of Miami Miller School of Medicine and from local community centers from Miami-Dade County from March 2010 to October 2011. Thirty-six participants failed the screening inclusion/exclusion criteria, 24 participants were eligible for the study, but never enrolled, and 60 eligible participants were enrolled in the study at baseline.

### 2.2. Study Design

#### 2.2.1. Inclusion and Exclusion Criteria

Potential study candidates were identified as individuals who expressed an interest in a study assessing the efficacy of a dietary supplement on depression, anxiety, and quality of life. Subjects were enrolled if they were (a) 18 years of age and older; (b) currently diagnosed with MDD or a related depressive disorder as classified by the DSM-IV-TR; (c) English speaking; (d) had an elevated level of homocysteine (>10 *μ*mol/L) at screening as a marker of inflammation; (e) interested in participating in a novel nutritional supplement program; and (f) willing to follow recommendations, including discontinuing all dietary supplements (e.g., multivitamin and mineral formula and vitamin B complex) for depression 2 weeks before starting and during the entire intervention period. Exclusion criteria consisted of (a) current enrollment in another research trial for depression treatment; (b) inability to consent to the study; or (c) pregnancy in women.

#### 2.2.2. Screening

Potential study subjects were prescreened for the inclusion and exclusion criteria and given a brief introduction to the nature and purpose of the study. All otherwise eligible subjects had a sample of venous blood drawn to measure homocysteine [16], a sulfurated amino acid derived from methionine, with higher levels directly related to depression. The blood sample was collected in an EDTA tube and delivered to the laboratory for processing within 2 hours of collection.

#### 2.2.3. Baseline Assessment and Randomization

Participants passing the screening process (including an elevated level of homocysteine >10 *μ*mol/L) were enrolled in the study and administered the baseline assessment. Following completion of the baseline assessment, participants were randomly assigned to one of two conditions (a) Max Stress B (a whole-food dietary supplement) or (b) placebo. Assignment of subjects into one of the two treatment groups was accomplished with a computer-generated table of random permutations, designed to balance the number of subjects in each group. The table was arranged in advance, and the predetermined list of treatments served to prepare the numbered supplement containers (used in order) and the envelopes to be opened in the case of emergency. All subjects and investigators were blind to the treatment condition. Only the staff at Premier Research Labs (the manufacturer of Max Stress B) knew the assignment of treatment condition.

#### 2.2.4. Outcomes and Assessments

Each participant completed a basic sociodemographics and medical history questionnaire, including current medications, at baseline. They were also asked to note any changes in type or amount of medications during the course of the study. Criteria used to select the assessment instruments included (a) appropriateness for the population; (b) ease of administration and scoring; (c) experience administering these measures; and (d) employment of measures involving a multimethod (i.e., self-report and physical measures) approach to enhance the validity of the overall assessment.

#### 2.2.5. Symptoms of Depression and Anxiety

The Beck Depression Inventory-II (BDI) [17] and Beck Anxiety Inventory (BAI) [18] were administered at baseline and 30- and 60-day followups to assess changes in depressive and anxiety symptoms, respectively, over the course of the intervention. Both the BDI and BAI consist of 21 items and are scored 0 to 63, where higher levels indicate progressive levels of depression (0–9: minimal, 10–18: mild, 19–29: moderate, and 30–63: severe) and anxiety (0–7: minimal, 8–15: mild, 16–25: moderate, and 26–63: severe).

#### 2.2.6. Quality of Life

The secondary outcome, administered at baseline and 30- and 60-day followups, included change in general health-related quality of life according to the Medical Outcomes Study Short Form 36 (SF-36) [19]. The SF-36 provides psychometrically based physical and mental health summary measures and as such is sensitive to subtle changes in relatively healthy persons, including those due to illness or injury. In addition, the SF-36 is reliable, valid, and provides a t score for each scale or domain ranging from 0 to 100 with higher scores representing better perceived quality of life.

#### 2.2.7. Physical Activity Level

As a control variable, physical activity was assessed at baseline and 30- and 60-day followups by the Stanford 7-Day Physical Activity Recall. This instrument has been validated for use in community-based settings, and it assesses the amount (number of hours) of moderate, physically challenging, and very physically challenging activities over the past 7 days. This assessment tool provides useful estimates of habitual physical activity for research and highly correlates with daily self-report of physical activity [20, 21].

#### 2.2.8. Intervention

For the 60-day intervention period, participants who enrolled in the study received (a) Max Stress B (a whole nutrient natural source extract from probiotic colonies that contains vitamins B1, B2, B3, B5, B6, and B12, and folate, PABA, biotin, inositol, purified water, and certified organic alcohol) or (b) placebo (an oil/water emulsion with food coloring similar to the test product). Subjects were instructed to consume 1 vial (equivalent to 1/2 teaspoon) of product in at least 12 ounces of water over the course of each day. They were not advised to modify eating or physical activity habits or nondepression prescription medication use. Subjects were also instructed not to consume other nutritional supplements containing any of the vitamin B complex nutrients, SAMe, inositol, PABA, or folate for two weeks prior to having the baseline assessment and until the conclusion of the 60-day intervention period. All study participants were compensated $40 for completing each of the three assessments.

#### 2.2.9. Statistical Analysis

Data were analyzed using SPSS 19 (IBM Inc., Chicago, IL, USA) for Windows. Frequency and descriptive statistics were calculated on all variables. Analysis of variance and Chi-square were utilized to determine the presence of differences in background contextual variables by study arm assignment. We utilized linear mixed modeling (LMM) to assess the fixed effect of time by randomization (Max Stress B versus placebo) on changes in our outcome variables from baseline to 60-day followup. If the type III test of the fixed effect of time by randomization was significant, then we used pairwise comparisons to determine the unique differences in effects over time by study arm between baseline and followup at 30 and 60 days for depression, anxiety, and quality of life variables. LMM with heterogeneous compound symmetry covariance allowed us to account for subject attrition, intercorrelated responses between time points, and nonconstant variability. The criterion for statistical significance was *α* = 0.05.

## 3. Results

### 3.1. Safety and Tolerability

During the entire study period, no subjects reported adverse events or complications from the test products.

### 3.2. Sociodemographics, Health Risk, Medication Use, and Physical Activity

Table 1 presents the sociodemographic variables by study arm assignment for age, gender, race/ethnicity, education, and marital status. The sample (*n* = 60) comprised of 68% males (*n* = 41) and 32% females (*n* = 19) with a mean age of 51 years (SD = 7.8;  *R* = 22,68). The racial/ethnic distributions of the subjects were as follows: 63% black, non-Hispanic (*n* = 38), 20% Hispanic (*n* = 12), and 16.7% white, non-Hispanic (*n* = 10).  Table 2 shows the most commonly prevalent history of diseases and disorders among this sample, including hypertension, arthritis, hepatitis, low back pain/herniated disc, and sleep apnea. None of these conditions was significantly different between study arms.  Table 3 displays the most prevalent current prescription medications and over-the-counter remedies, including antianxiety, antidepressant, antiviral, antihypertensive, and insomnia and aspirin, Tylenol, and vitamin/mineral dietary supplements. No proportions were significantly different between the study groups. Regarding physical activity, study groups were statistically similar on the number of times exercised at strenuous, moderate, and mild exertion levels in the previous 7 days at each assessment.

### 3.3. Depression and Anxiety

**Figure 1:**
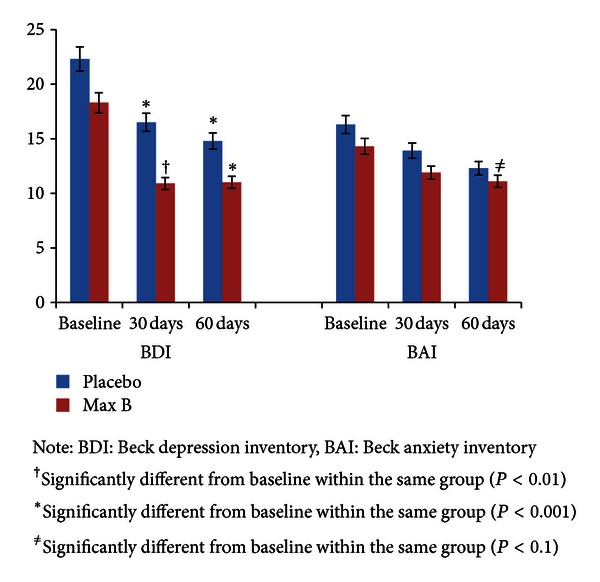
Depression and anxiety at baseline, 30 days, and 60 days.

Figure 1 shows the mean values for the BDI and BAI over the course of the intervention for both study groups. For the BDI, the fixed effects for time (*F*[2,83.3] = 21.7, *P* < 0.01) and randomization (*F*[1,57.6] = 4.0, *P* = 0.05) were significant, but the effect for time by randomization was nonsignificant. Post hoc comparisons revealed that the BDI significantly decreased from baseline to 30 days (mean difference = 6.8; SE = 1.2; 95% CI: 3.8, 9.7; *P* < 0.01) and 60 days (mean difference = 7.7; SE = 1.3; 95% CI: 4.5, 10.9; *P* < 0.01) for the total sample. For the Max Stress B group, the BDI significantly decreased from baseline to 30 days (mean difference = 5.8; SE = 1.7; 95% CI: 1.7, 10.0; *P* < 0.01) and 60 days (mean difference = 7.5; SE = 1.8; 95% CI: 3.1, 12.0; *P* < 0.001). For the placebo group, the BDI significantly decreased from baseline to 30 days (mean difference = 7.7; SE = 1.7; 95% CI: 3.4, 11.9; *P* < 0.001) and 60 days (mean difference = 7.9; SE = 1.9; 95% CI: 3.2, 12.5; *P* < 0.001). For the BAI, the fixed effect for time (*F*[2,93.4] = 3.8, *P* < 0.05) was significant, but the effects for randomization and time by randomization were nonsignificant. Post hoc comparisons revealed that the BAI significantly decreased from baseline to 60 days (mean difference = 3.8; SE = 1.4; 95% CI: 0.4, 7.2; *P* < 0.05) for the total sample. The BAI showed a positive trend, but was statistically not significant, from baseline to 60 days (mean difference = 4.2; SE = 1.9; 95% CI: −0.5, 8.8; *P* = 0.10) for the Max Stress B group, whereas the placebo group's score stayed flat over the course of the intervention (mean difference = 3.4; SE = 2.0; 95% CI: −1.4, 8.3; *P* = 0.25).

### 3.4. Quality of Life

Table 4 shows the descriptive values for the SF-36, including the scores for physical functioning, role-physical, general health, vitality, social functioning, role-emotional, mental health, and bodily pain. For role-physical, general health, and bodily pain the fixed effects for time, randomization, and time by randomization were nonsignificant. For physical functioning, the time by randomization effect was not significant (*F*[2,87.9] = 3.0, *P* = 0.06), and post hoc comparisons revealed that the 60-day score was significantly higher than the baseline (mean difference = 11.6; SE = 4.6; 95% CI: 0.3, 22.9; *P* < 0.05) for the placebo group. For vitality, the fixed effect for time (*F*[2,78.9] = 5.6,  *P* < 0.01) was significant, but the effects for randomization and time by randomization were nonsignificant. Post hoc comparisons revealed that the score significantly increased from baseline to 30 days (mean difference = 6.3; SE = 2.5; 95% CI: 0.3, 12.3; *P* < 0.05) and 60 days (mean difference = 8.3; SE = 2.7; 95% CI: 1.7, 14.8; *P* < 0.01) for the total sample. For social functioning, the fixed effect for time by randomization (*F*[2,85.1] = 2.5,  *P* = 0.09) was not significant, and the effect for time (*F*[2,85.1] = 5.9,  *P* < 0.01) was significant, but the effect for randomization was nonsignificant. Post hoc comparisons revealed that the placebo group score was significantly higher than the Max Stress B group (mean difference = 16.6; SE = 6.6; 95% CI: 3.3, 29.9; *P* < 0.05) at 30 days. For the placebo group, the score at 30 days (mean difference = 14.8; SE = 4.5; 95% CI: 3.9, 25.7; *P* < 0.01) and 60 days (mean difference = 11.3; SE = 4.7; 95% CI: −0.1, 22.8; *P* = 0.05) significantly improved compared to baseline. For role-emotional, the fixed effect for time (*F*[2,77.4] = 5.3, *P* < 0.01) was significant, but the effects for randomization and time by randomization were nonsignificant. Post hoc comparisons revealed that the score significantly increased from baseline to 30 days (mean difference = 10.3; SE = 3.6; 95% CI: 1.4, 19.2; *P* < 0.05) and 60 days (mean difference = 10.3; SE = 3.9; 95% CI: 0.7, 19.8; *P* < 0.05) for the total sample. For mental health, the fixed effects for time by randomization (*F*[2,92.1] = 3.6,  *P* < 0.05) and time (*F*[2,92.1] = 13.6,  *P* < 0.01) were significant, but the effect for randomization was nonsignificant. Post hoc comparisons revealed that the placebo group score was significantly higher than the Max Stress B group (mean difference = 13.9; SE = 6.2; 95% CI: 1.5, 26.2; *P* < 0.05) at 30 days. For the Max Stress B group, the scores at 30 days (mean difference = 9.5; SE = 3.5; 95% CI: 0.9, 18.2; *P* < 0.05) and 60 days (mean difference = 15.8; SE = 3.4; 95% CI: 7.6, 24.0; *P* < 0.01) were significantly improved compared to baseline. For the placebo group, the score at 30 days (mean difference = 13.0; SE = 3.6; 95% CI: 4.2, 21.8; *P* < 0.01) significantly improved, but not at 60 days.

## 4. Discussion

In the current study, we have demonstrated moderate short-term (60 days) improvements in depression, anxiety, and overall mental health with the Max Stress B product, which contains several whole-food nutrients. Globally, the BDI and BAI are valid and reliable assessments used to detect many symptoms of depression and anxiety, respectively [17, 18], and both study groups demonstrated improved scores from baseline on both assessment tools. The Max Stress B group showed greater improvement on the BAI, while the placebo group demonstrated greater improvement on the BDI (25% versus 22% and 34% versus 39%, respectively). However, the Max Stress B group achieved a more continuous decrease throughout the protocol, while the placebo group had less or no improvement from 30 to 60 days. Considering the positive trend demonstrated by the Max Stress B group, our study may not have been of sufficient length to demonstrate even more significant improvements. Thus, our findings are modestly similar to those of others showing that dietary supplements have the ability to improve certain aspects of mood [11–15].

In addition to some mood benefits, the Max Stress B arm showed positive effects on the mental health scale according to the SF-36. Our findings are consistent with another study that showed improvements in mood (depression and anxiety) along with memory, concentration, and fatigue in response to consuming Ginkgo biloba [13]. Another recent study found that Ginkgo biloba was beneficial on scores of neuropsychiatric impairment, including apathy and depression/dysphoria, compared to placebo in a sample of adults with dementia [22]. An additional study showed some improvement in ratings of depression and cognition after taking Ginkgo biloba in a sample of adults who had Alzheimer's [23]. Thus, our findings appear to support other research that Max Stress B, similar to other nutrients, has the ability to improve subjective ratings of mental health.

In summary, we have showed that Max Stress B offers utility for improving the overall mental health quality of life of adults with MDD or another depressive disorder with no side effects. Our findings are somewhat consistent with the work of others in the depression literature. Furthermore, future trials should include longer interventions to more firmly determine the effect of Max Stress B on mental health and mood of adults suffering from depressive disorders.

### 4.1. Limitations

We note several limitations of the current investigation. We enrolled English speaking individuals only, so our results may not be generalizable to non-English speaking persons of different racial/ethnic backgrounds. Our findings may be restricted by the length of the intervention, given that mood disorder symptom changes may take longer than 60 days to occur. We did not assess dietary intake; thus, we were not able to control for possible influences that variable may have had on our final results. However, we did assess physical activity levels, which were found to be unrelated to the outcome variables. Furthermore, we did not restrict or change the use of medications by our participants, such as the use of steroids, antidepressants, or anti-inflammatory agents, given the ethical considerations associated with such decision. The findings of our study are also potentially limited by a small sample size in each study arm. A larger sample size could result in even more significant findings for mood symptoms and quality of life. Finally, we did not assess homocysteine at the end of the trial. Assessing the changes in homocysteine and depression and anxiety scores could have provided useful information and would be of interest in future trials.

## 5. Conclusions

Depression is a significant problem that is increasing in prevalence. In fact, approximately 15 million adults have MDD, and the prevalence of MDD and its associated financial costs are a significant drain on an already overburdened United States health system and are getting worse [1]. This disease shows signs of spiraling out of control, as options for prevention or treatment are limited. Thus, any safe intervention that demonstrates promise for either sustaining mood or improving the condition of persons with MDD or another depressive disorder is urgently needed.

The formula used in the current study was well tolerated among all subjects. The Max Stress B formula showed modest improvements in mood and mental health according to the BDI, BAI, and SF-36, making our findings consistent with the prior studies. Thus, our study shows that a high quality, whole-food dietary supplement may offer an opportunity for adults with depression to improve mood symptoms and quality of life.

## Figures and Tables

**Table 1:** Sociodemographic characteristics of the sample.

Variable	Category	Total Sample (*n* = 60)	Max Stress B (*n* = 30)	Placebo (*n* = 30)	Statistic
Age	—	*M* = 50.9 (SD = 7.8; *R* = 22, 68)	*M* = 49.3 (SD = 8.1; *R* = 22, 68)	M = 52.5 (SD = 7.3; *R* = 29, 67)	*t* (58) = 1.6, *P* = 0.11

Gender	Male	41 (68.3%)	19 (63.3%)	22 (73.3%)	*χ* ^2^(1) = 0.4, *P* = 0.69
	Female	19 (31.7%)	11 (36.7%)	8 (26.7%)	

Race/ethnicity	White, non-Hispanic	10 (16.7%)	4 (13.3%)	6 (20.0%)	*χ* ^2^(2) = 0.5, *P* = 0.78
	Black, non-Hispanic	38 (63.3%)	20 (66.7%)	18 (60.0%)	
	Hispanic	12 (20.0%)	6 (20.0%)	6 (20.0%)	

Education	Up to high school	40 (67.8%)	22 (73.3%)	18 (62.1%)	*χ* ^2^(3) = 4.5, *P* = 0.22
	Some post high school training	11 (18.6%)	6 (20.0%)	5 (17.2%)	
	College graduate	4 (6.8%)	—	4 (13.8%)	
	Master's degree or higher	4 (6.8%)	2 (6.7%)	2 (6.9%)	

Marital status	Never married	29 (49.2%)	14 (46.7%)	15 (51.7%)	*χ* ^2^(3) = 0.5, *P* = 0.92
	Married	7 (11.9%)	4 (13.3%)	3 (10.3%)	
	Widowed	3 (5.1%)	2 (6.7%)	1 (3.4%)	
	Divorced/separated	20 (33.9%)	10 (33.4%)	10 (34.5%)	

Note: *M*: mean; SD: standard deviation; and *R*: range.

**Table 2:** Prevalence of diseases and disorders.

Disease/disorder	Category	Total Sample	Max Stress B	Placebo	Statistic
Hypertension	Yes	26 (44.1%)	11 (36.7%)	15 (51.7%)	χ^2^(1) = 1.4, *P* = 0.24
	No	33 (55.9%)	19 (63.3%)	14 (48.3%)	

Arthritis	Yes	8 (13.6%)	4 (13.3%)	4 (13.8%)	χ^2^(1) = 0.01, *P* = 0.96
	No	51 (86.4%)	26 (86.7%)	25 (86.2%)	

Hepatitis	Yes	11 (18.6%)	7 (23.3%)	4 (13.8%)	χ^2^(1) = 0.9, *P* = 0.35
	No	48 (81.4%)	23 (76.7%)	25 (86.2%)	

Low back pain/herniated disc	Yes	17 (28.8%)	10 (33.3%)	7 (24.1%)	χ^2^(1) = 0.6, *P* = 0.44
	No	42 (71.2%)	20 (66.7%)	22 (75.9%)	

Sleep apnea	Yes	8 (13.6%)	2 (6.7%)	6 (20.7%)	χ^2^(1) = 2.5, *P* = 0.12
	No	51 (86.4%)	28 (93.3%)	23 (79.3%)	

**Table 3:** Prevalence of prescription and over-the-counter medication usage.

Medication		Category	Total Sample	Max Stress B	Placebo	Statistic
	Antianxiety	Yes	9 (15.3%)	3 (10.0%)	6 (20.7%)	*χ* ^2^(1) = 1.3, *P* = 0.25
		No	50 (84.7%)	27 (90.0%)	23 (79.3%)	
	Antidepressant	Yes	26 (44.1%)	16 (53.3%)	10 (34.5%)	*χ* ^2^(1) = 2.1, *P* = 0.15
		No	33 (55.9%)	14 (46.7%)	19 (65.5%)	
Current prescription	Antiviral	Yes	10 (16.9%)	4 (13.3%)	6 (20.7%)	*χ* ^2^(1) = 0.6, *P* = 0.45
		No	49 (83.1%)	26 (86.7%)	23 (79.3%)	
	Antihypertensive	Yes	10 (16.9%)	3 (10%)	7 (24.1%)	*χ* ^2^(1) = 2.1, *P* = 0.15
		No	49 (83.1%)	27 (90%)	22 (75.9%)	
	Insomnia	Yes	19 (32.2%)	11 (36.7%)	8 (27.6%)	*χ* ^2^(1) = 0.6, *P* = 0.46
		No	40 (67.8%)	19 (63.3%)	21 (72.4%)	

	Aspirin	Yes	11 (18.6%)	4 (13.3%)	7 (24.1%)	*χ* ^2^(1) = 1.1, *P* = 0.29
		No	48 (81.4%)	26 (86.7%)	22 (75.9%)	
OTC in the prior week	Tylenol	Yes	13 (22%)	7 (23.3%)	6 (20.7%)	*χ* ^2^(1) = 0.1, *P* = 0.81
		No	46 (78%)	23 (76.7%)	23 (79.3%)	
	Vitamin/mineral	Yes	10 (16.9%)	3 (10%)	7 (24.1%)	*χ* ^2^(1) = 2.1, *P* = 0.15
		No	49 (83.1%)	27 (90%)	22 (75.9%)	

**Table 4:** Physical and mental functioning on the SF-36 at baseline, 30 days, and 60 days.

Measure	Time	Total Sample	Max Stress B	Placebo
	Baseline	54.2 ± 28.1 (0, 100)	56.2 ± 29.6 (5, 100)	52.1 ± 26.7 (0, 95)
Physical functioning	30 Days	56.4 ± 25.8 (5, 100)	54.5 ± 27.1 (5, 100)	58.4 ± 24.7 (10, 100)
	60 Days	56.5 ± 28.4 (5, 100)	51.4 ± 28.3 (5, 100)	62.4 ± 27.8 (10, 100)*

	Baseline	47.8 ± 27.2 (0, 100)	47.3 ± 29.5 (0, 100)	48.3 ± 25.2 (0, 93.75)
Role-physical	30 Days	51.8 ± 28.1 (0, 100)	45.9 ± 28.9 (0, 100)	57.8 ± 26.4 (18.75, 100)
	60 Days	51.9 ± 28.5 (0, 100)	49.1 ± 29.5 (0, 100)	55.0 ± 27.5 (0, 100)

	Baseline	57.8 ± 22.4 (10, 100)	59.0 ± 24.9 (10, 100)	56.5 ± 19.9 (20, 92)
General health	30 Days	59.3 ± 22.7 (10, 100)	57.6 ± 23.6 (10, 100)	61.1 ± 22.0 (10, 92)
	60 Days	59.4 ± 26.2 (0, 100)	59.8 ± 28.6 (0, 100)	59.0 ± 23.6 (15, 97)

	Baseline	47.5 ± 18.5 (0, 93.75)	45.0 ± 19.5 (6.25, 93.75)	50.0 ± 17.4 (0, 81.25)
Vitality	30 Days	53.6 ± 20.4 (6.25, 100)*	52.8 ± 20.3 (18.75, 100)	54.5 ± 20.9 (6.25, 100)
	60 Days	55.7 ± 22.9 (6.25, 100)^*≠*^	56.9 ± 21.4 (18.75, 100)	54.3 ± 24.9 (6.25, 100)

	Baseline	50.9 ± 24.8 (0, 100)	49.2 ± 24.1 (0, 100)	52.6 ± 25.7 (0, 100)
Social functioning	30 Days	59.0 ± 26.3 (0, 100)	51.3 ± 27.0 (0, 100)	67.0 ± 23.4 (25, 100)^*≠*†^
	60 Days	60.7 ± 25.3 (0, 100)	59.1 ± 25.6 (0, 100)	62.5 ± 25.3 (12.5, 100)*

	Baseline	47.0 ± 25.6 (0, 100)	47.8 ± 29.1 (0, 100)	46.3 ± 21.8 (0, 91.7)
Role-emotional	30 Days	56.4 ± 29.9 (0, 100)*	50.0 ± 31.1 (0, 100)	63.1 ± 27.6 (16.67, 100)
	60 Days	56.0 ± 30.9 (0, 100)*	52.6 ± 31.0 (0, 100)	60.0 ± 31.0 (0, 100)

	Baseline	51.8 ± 21.9 (0, 100)	46.7 ± 21.8 (0, 100)	57.1 ± 21.1 (0, 90)
Mental health	30 Days	62.3 ± 24.3 (5, 95)	55.2 ± 26.7 (5, 95)*	69.6 ± 19.4 (25, 95)^†^
	60 Days	61.9 ± 20.9 (5, 100)	61.6 ± 21.6 (5, 100)^*≠*^	62.2 ± 20.5 (30, 100)

	Baseline	51.1 ± 24.7 (0, 90)	50.1 ± 23.8 (10, 90)	52.0 ± 26.0 (0, 90)
Bodily pain	30 Days	55.5 ± 25.8 (0, 90)	52.7 ± 24.9 (0, 90)	58.4 ± 26.8 (12, 90)
	60 Days	56.4 ± 25.7 (0, 90)	53.9 ± 27.9 (0, 90)	59.2 ± 23.2 (22, 90)

Note: Values are mean ± standard deviation (minimum, maximum).

*Significantly different from baseline within the same group (*P* < 0.05).

^*≠*^Significantly different from baseline within the same group (*P* < 0.01).

^†^Significantly different from Max Stress B at the same time point (*P* < 0.05).
